# Strategies for Optimizing Water-Exchange Rates of Lanthanide-Based Contrast Agents for Magnetic Resonance Imaging

**DOI:** 10.3390/molecules18089352

**Published:** 2013-08-05

**Authors:** Buddhima N. Siriwardena-Mahanama, Matthew J. Allen

**Affiliations:** Department of Chemistry, Wayne State University, Detroit, MI 48202, USA

**Keywords:** magnetic resonance imaging, contrast agents, PARACEST, water-exchange rate

## Abstract

This review describes recent advances in strategies for tuning the water-exchange rates of contrast agents for magnetic resonance imaging (MRI). Water-exchange rates play a critical role in determining the efficiency of contrast agents; consequently, optimization of water-exchange rates, among other parameters, is necessary to achieve high efficiencies. This need has resulted in extensive research efforts to modulate water-exchange rates by chemically altering the coordination environments of the metal complexes that function as contrast agents. The focus of this review is coordination-chemistry-based strategies used to tune the water-exchange rates of lanthanide(III)-based contrast agents for MRI. Emphasis will be given to results published in the 21st century, as well as implications of these strategies on the design of contrast agents.

## 1. Introduction

This review focuses on coordination-chemistry-based strategies from the past decade used to tune the water-exchange rates of lanthanide-based *T*_1_-shortening and paramagnetic chemical exchange saturation transfer (PARACEST) contrast agents for magnetic resonance imaging (MRI) as well as the implications of these strategies on the development of new contrast agents. MRI is a non-invasive imaging modality that is widely used in clinical medicine and biomedical research to generate three-dimensional images with a high spatial resolution (~1 mm^3^) and excellent tissue penetration [1]. Conventional MRI generates contrast through differences in water proton density and the longitudinal and transverse relaxation times of water protons. These differences provide information regarding the chemical and physical nature of an imaged specimen. However, a key challenge associated with MRI is the low inherent contrast that is often observed [2]. To overcome this challenge, paramagnetic substances known as contrast agents are used to catalytically shorten the longitudinal (*T*_1_) and transverse (*T*_2_) relaxation times of nearby water protons [2,3,4]. This shortening leads to improved image contrast between regions that differ in the amount of contrast agent present. Contrast agents that shorten both longitudinal and transverse relaxation times to approximately the same degree are called *T*_1_-shortening agents. Agents that shorten transverse relaxation times to a much greater extent than longitudinal relaxation times are called *T*_2_-shortening agents. In general, *T*_1_-shortening agents give rise to increased signal intensity and are referred to as positive contrast agents, and *T*_2_-shortening agents give rise to a decreased signal intensity and are referred to as negative contrast agents [5]. Of these two types of agents, *T*_1_-shortening agents are often favored because the darker images produced by *T*_2_-shortening agents can be difficult to differentiate from background [6]. Consequently, this review will focus on *T*_1_-shortening agents.

The most widely used clinical contrast agents are Gd^III^-containing acyclic or macrocyclic polyaminopolycarboxylate-based chelates. The efficiency of these Gd^III^-containing complexes as contrast agents is described by their ability to shorten the relaxation times of nearby water protons and is expressed as relaxivity, *r*_1_, with units of mM^−1^ s^−1^. The relaxivity of Gd^III^-based contrast agents is governed by magnetic field strength and several structural, dynamic, and electronic parameters of the agents including the number of coordinated water molecules, *q*; the rate of exchange between coordinated and bulk water, *k_ex_*; the distance between the Gd^III^ ion and coordinated water protons, *r_GdH_*; the longitudinal and transverse electron spin relaxation times *T*_1*e*_ and *T*_2*e*_, respectively; and the rotational correlation time of the agent, *τ_R_*. The contributions of these molecular parameters to relaxivity are described by the Solomon–Bloembergen–Morgan (SBM) equations, and have been discussed in detail elsewhere [7,8,9]. 

**Figure 1:**
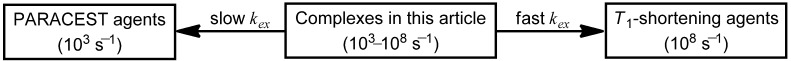
Optimum water-exchange rates of *T*_1_ and PARACEST agents.

Based on the SBM equations, an optimum relaxivity of approximately 40 mM^−1^ s^−1^ per coordinated water molecule is expected at the clinically relevant field strength of 1.5 T for Gd^III^-containing contrast agents [8,9]. However, the observed relaxivity of clinically approved contrast agents is much lower (~4–5 mM^−1^ s^−1^) than the theoretical optimum value [10]. The low observed relaxivity suggests that the molecular parameters that govern the relaxivity of these agents need to be tuned. A great deal of research has been employed to tune the molecular parameters that influence relaxivity using coordination chemistry. For example, relaxivity increases as a function of water-coordination number, and water-coordination number is a molecular parameter that can be tuned through modification of ligands. However, the tuning of water-coordination number is limited by issues beyond relaxivity: increasing water-coordination number beyond two often compromises complex stability, which has a detrimental impact on toxicity. Another molecular parameter that can be tuned using coordination chemistry is rotational correlation time, where slow rotation leads to high relaxivity. Attempts to slow rotation through interactions with high-molecular weight species have been successful, but the maximum effect of high molecular weight interactions are not observed due to internal motion, slow water-exchange rates, or both. Therefore, tuning water-exchange rate becomes crucial in designing efficient *T*_1_-shortening agents (Figure 1). 

The optimum water-exchange rate for small-molecular *T*_1_-shortening agents is ~10^8^ s^−1^ at 1.5 T, based on the SBM equations, and this rate is roughly two orders of magnitude faster than the water-exchange rates of current clinical agents (~10^6^ s^−1^) [8,9,10]. It is also important to note that water exchange provides the lower limit for proton exchange, but at physiologically relevant pH values, proton and water exchange are roughly the same [9,11]. Consequently, this review focuses on water-exchange rates and not proton-exchange rates.

In addition to *T*_1_-shortening agents, a relatively new class of contrast agents that function based on the transfer of magnetization by chemical exchange of protons has gained much attention in the last two decades. These agents are referred to as chemical exchange saturation transfer (CEST) agents. One limitation of CEST agents is the small signal produced from direct presaturation of bulk water that occurs coincidentally during presaturation of exchangeable protons. This limitation can be overcome with the use of paramagnetic lanthanide(III) (Ln^III^)-containing complexes (in this article, Ln^III^ = Ce^III^, Pr^III^, Nd^III^, Sm^III^, Eu^III^, Tb^III^, Dy^III^, Ho^III^, Er^III^, Tm^III^, or Yb^III^) that are able to shift the resonance frequency of exchangeable protons away from that of bulk water, and these complexes are called paramagnetic chemical exchange saturation transfer (PARACEST) contrast agents. Reviews of PARACEST agents in general can be found elsewhere [12,13,14]. The exchangeable protons on PARACEST agents can be O–H or N–H protons on a multidentate ligand or protons from Ln^III^-coordinated water molecules that undergo chemical exchange with bulk water. This review focuses on water-exchange-based agents. 

PARACEST agents give rise to a decrease in the bulk water magnetization as a result of the transfer of saturated magnetization from exchangeable protons (coordinated water molecules for the purpose of this review) to bulk water after selective presaturation of the coordinated-water protons. The efficiency of PARACEST agents is measured in terms of the magnitude of percent saturation transfer. For transfer of saturated magnetization to occur, the difference in resonance frequency between the two exchanging pools of protons, ∆*ω*, needs to be greater than or equal to the rate of exchange between the two pools of exchanging protons, *k_ex_* [15]. Paramagnetic Ln^III^-containing complexes can display ∆*ω* values that are about an order of magnitude greater than those observed for diamagnetic systems that undergo magnetization transfer [12,16]. Large values of ∆*ω* are advantageous because they enable selective presaturation of exchangeable protons without direct saturation of bulk water. For ∆*ω* to be greater than or equal to the *k_ex_*, PARACEST agents require slow water-exchange rates (~10^3^ s^−1^) to maximize efficiency (Figure 1). However, most PARACEST agents developed to date display water-exchange rates that are about an order of magnitude faster (~10^4^ s^−1^) than the optimum water-exchange rate [17]. 

Tuning water-exchange rate is of great importance to achieve maximum efficiencies for both *T*_1_-shortening and PARACEST agents; however, these classes of agents require tuning in opposite directions: *T*_1_-shortening agents require fast rates and PARACEST agents require slow rates. The need to tune water-exchange rates over a broad range from slow (~10^3^ s^−1^) to fast (~10^8^ s^−1^) has led to an enormous focus on coordination-chemistry-based strategies to tune and on analytical techniques to measure the rates and mechanisms of exchange. Determination of the rates and mechanisms of water exchange of Ln^III^-containing complexes has provided a general understanding of the influence of the structure of Ln^III^-containing complexes on water-exchange rates. The water-exchange rates of Ln^III^-containing complexes are often determined from the temperature-dependence of the transverse relaxation rates of ^17^O-enriched water measured by variable-temperature ^17^O-NMR spectroscopy. The mechanism of water exchange is determined from the volume of activation (∆*V*^‡^) obtained from the pressure-dependence of the transverse relaxation rate of ^17^O-enriched water determined using variable-pressure ^17^O-NMR spectroscopy. Experimental details for these measurements have been described elsewhere [18,19,20] and, therefore, are not included in this review. Because water-exchange rates depend on temperature, comparisons made among water-exchange rates in this article are only between rates measured at the same temperature. All water-exchange rates reported in the text have been determined at 25 °C unless otherwise noted. 

The importance and determinants of water-exchange rate of Gd-based *T*_1_-shortening agents were reviewed at the end of the last century [21]. In addition, the importance of water-exchange rates was reviewed recently with respect to responsive *T*_1_-shortening and PARACEST agents [22]. The following text describes coordination-chemistry-based strategies explored over the last decade to tune the water-exchange rates of *T*_1_-shortening and PARACEST agents and the implications of these strategies for improving the efficiency of both types of contrast agents. 

## 2. Coordination-Chemistry-Based Strategies to Tune *k_ex_*

Coordination-chemistry-based strategies that have been used to tune the water-exchange rates of Ln^III^-containing complexes for *T*_1_-shortening and PARACEST agents include modification of (1) the mechanism of water exchange; (2) the charge of the Ln^III^-containing complex; (3) the steric hindrance at the site of water coordination; (4) the ligand side chains; and (5) the ratio of twisted-square-antiprism (TSAP) to square-antiprism (SAP) isomers for 1,4,7,10-tetraazacyclododecane-1,4,7,10-tetraacetate (DOTA)-type complexes. In addition to the five strategies listed above, the identity of the Ln^III^ ion in a complex also influences the magnitude of water-exchange rates [23,24,25,26,27]. However, this review focuses on strategies useful to both *T*_1_-shortening and PARACEST agents, and therefore, a description of the influence of Ln^III^ ion on water-exchange rates is not included because Ln^III^ ions other than Gd^III^ are not useful for *T*_1_-shortening agents. The following text is divided into separate sections that describe the five strategies listed above in terms of their implications to *T*_1_-shortening and PARACEST agents. Although these strategies are separated in this text to enable structure–function comparisons to be made, it is important to note that these strategies are interrelated and cannot be completely isolated from each other.

### 2.1. Modification of Water-Exchange Mechanism

**Figure 2:**
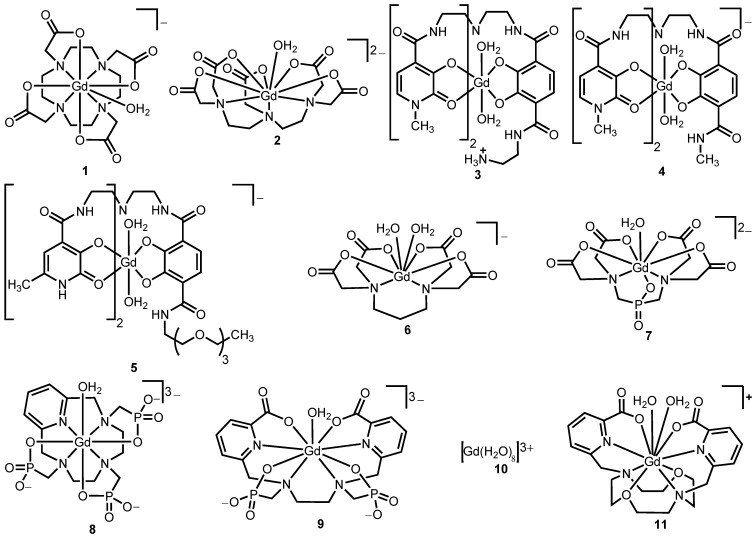
Representative Gd^III^-containing complexes that undergo water exchange via dissociative (**1** and **2**), dissociative interchange (**9**), associative (**3**, **4**, **7**, **8**, **10**, and **11**), and associative interchange (**5** and **6**) processes [28,29,30,31,32,33,34,35,36,37].

The mechanism of water exchange is an important determinant of the magnitude of water-exchange rates of Ln^III^-containing complexes. In this section, the relationship between the mechanism of water exchange and water-exchange rate will be discussed using complexes **1**–**11** (Figure 2 and Table 1). Water exchange in Ln^III^-containing complexes proceeds via dissociative, associative, or interchange mechanisms. In a dissociative mechanism, dissociation of the coordinated water is rate limiting and precedes coordination of the incoming water molecule. In an associative mechanism, coordination of the incoming water molecule is rate limiting and precedes the dissociation of the coordinated water molecule. The interchange mechanisms are where transition states are not observed. Interchange mechanisms are divided in to dissociative interchange and associative interchange mechanisms depending on the relative contributions from bond breaking and forming: higher bond-breaking contributions lead to dissociative interchange and higher bond-forming contributions lead to associative interchange. Ln^III^-containing complexes with coordination numbers of nine tend to undergo dissociative or dissociative interchange mechanisms of exchange, and complexes with coordination numbers of eight tend to undergo associative or associative interchange mechanisms of exchange. 

**Table 1:** Water-exchange parameters obtained from ^17^O-NMR spectroscopy and coordination numbers of complexes **1**−**11**.

Complex number	*k_ex_* (×10^6^ s^−1^)	∆*V*^‡^ (cm^3^ mol^−1^)	*q*	Coordination number	Mechanism	Reference
**1**	4.1	10.5	1	9	dissociative	[31]
**2**	3.3	12.5	1	9	dissociative	[31]
**2**	7.0 (37 °C)	nr	1	9	dissociative	[38]
**3**	380	nr	2	8	associative *^a^*	[30]
**4**	130	nr	2	8	associative *^a^*	[30]
**5**	53	ߝ5	2	8	associative interchange	[29]
**6**	102	–1.5	2	8	associative interchange	[33]
**7**	27	nr	1	8	associative *^a^*	[34]
**8**	170	nr	1	8	associative *^a^*	[32]
**9**	700	8.3	0.6	8 and 9	dissociative interchange	[36]
**10**	804	–3.3	8	8	associative	[31]
**11**	220	nr	1.4	9 and 10	associative *^a^*	[37]

nr = not reported, *^a^*associative, but no ∆*V*^‡^ data given.

**Figure 3:**
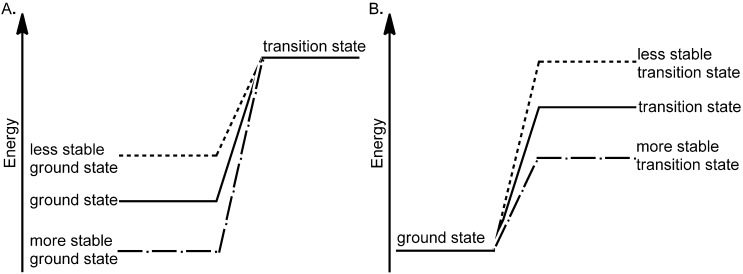
Ground and transition states of the water-exchange process. A. Stabilization and destabilization of the ground state lead to slower and faster water exchange-rates, respectively. B. Stabilization and destabilization of the transition state lead to faster and slower water-exchange rates, respectively.

In aqueous solution, Ln^III^ aqua ions usually have water-coordination numbers of eight or nine, with large lanthanides (La^III^ to Nd^III^) tending toward water-coordination number nine, medium-sized lanthanides (Sm^III^ to Gd^III^) existing mostly in equilibrium between water-coordination numbers eight and nine, and small lanthanides (Tb^III^ to Lu^III^) likely having water-coordination numbers of eight. Many polyaminopolycarboxylate-based ligands occupy eight coordination sites of Ln^III^ ions allowing space for the coordination of one water molecule. Because these types of complexes have coordinatively saturated (nine coordinate) ground states, they tend to undergo dissociative water exchange through a relatively unstable eight-coordinate transition state. Dissociative exchange often leads to slow water-exchange rates due to the large energy difference between the nine-coordinate ground state and the eight-coordinate transition state, and the rates of exchange could be increased by stabilizing the eight-coordinate transition state or destabilizing the nine-coordinate ground state. On the other hand, water-exchange rates could be decreased by stabilizing the nine-coordinate ground state or destabilizing the eight-coordinate transition state (Figure 3). 

Different from nine-coordinate complexes, eight-coordinate Ln^III^-containing complexes used as contrast agents tend to have six or seven coordination sites occupied by a multidentate ligand, enabling the coordination of one or two water molecules in the inner coordination sphere of the Ln^III^ ion. Due to the coordinatively unsaturated ground state, eight-coordinate complexes tend to undergo associative water exchange through a nine-coordinate transition state. Because the energy gaps between eight-coordinate ground states and nine-coordinate transition states tend to be small, associative exchange mechanisms often display fast water-exchange rates [28]. Therefore, with eight-coordinate complexes, stabilization of the nine-coordinate transition state is a potential method to increase water-exchange rates, and stabilization of the eight-coordinate ground state is likely to decrease water-exchange rates. 

Nine-coordinate polyaminopolycarboxylate complexes **1** and **2** display water-exchange rates on the order of 10^6^ s^−1^, and eight-coordinate complexes (compounds **3**–**8**) display relatively fast water-exchange rates (10^7^–10^8^ s^−1^). These differences in water-exchange rates can be attributed in part to the differences in the mechanism of water exchange. The water-exchange rates of hydroxypyridonate (HOPO)-based complexes **3**–**5**, reported by Raymond and co-workers, are closer to the optimum water-exchange rate (10^8^ s^−1^) of *T*_1_-shortening agents at 1.5 T than those of clinically approved polyaminopolycarboxylate-based complexes **1** and **2** [28,29,30,31]. Similar to HOPO-based complexes, the fast water-exchange rates observed for polyaminopolycarboxylate-based complexes **6** and **7** and phosphonate-containing complex **8** can be attributed in part to an associative exchange mechanism [32,33,34].

As mentioned earlier, water-exchange rates can be increased by stabilizing the transition state formed during the exchange process. This idea is exemplified by the 3-fold faster water-exchange rate of complex **3** relative to complex **4** [30]. This difference in water-exchange rate is likely due to the accommodation of a third water molecule in the inner-coordination sphere through intra-molecular hydrogen bonding between the primary amine of complex **3** and a third water molecule. Stabilization of the nine-coordinate transition state through intra-molecular hydrogen bonding is likely the cause of the faster water-exchange rate for complex **3** [30]. In another example, linear complex **9**, despite undergoing dissociative exchange, displays a water-exchange rate that is 210-times greater than the rate for linear complex **2** and is nearly as fast as the Gd^III^-aqua complex, **10**. This extremely fast water-exchange rate is likely due to the existence of both eight- and nine-coordinate species in solution as observed by variable temperature UV–vis spectroscopy of the Eu^III^ analog of complex **9**, implying that the eight-coordinate transition state is stabilized [35,36]. In a similar study, but on a macrocyclic complex, Tóth and co-workers demonstrated that macrocyclic complex **11** displayed a water-exchange rate that is 54-times faster than the rate of macrocyclic complex **1**, because of an equilibrium between two hydration states (*q* = 1 and *q* = 2) with coordination-numbers nine and ten based on variable temperature UV-vis spectroscopy. Complex **11** likely undergoes associative exchange based on the large negative activation entropy (–35 J mol^−1^ K^−1^); however, the authors did not report the volume of activation for complex **11** [37]. 

The fast water-exchange rates (10^7^–10^8^ s^−1^) of complexes **3**–**9** and **11** are desirable starting points for the design of *T*_1_-shortening agents. Designing eight-coordinate complexes with the ability to undergo associative exchange and complexes in which eight- and nine-coordinate species are in equilibrium with one another is desirable to attain fast water-exchange rates for *T*_1_-shortening agents. In addition to complexes that undergo associative exchange, complexes that undergo dissociative exchange, but with stable eight-coordinate transition states are likely to display fast water-exchange rates desirable for *T*_1_-shortening agents. However, stabilizing the eight-coordinate transition state can be challenging for most polyaminopolycarboxylate-based ligands because these ligands tend to favor complexes with coordination number nine. On the other hand, the fast water-exchange rates of complexes **3**–**9** and **11** and the relatively slower exchange rates (10^6^ s^−1^) of nine-coordinate polyaminopolycarboxylate-based complexes **1** and **2** are orders of magnitude too fast to be useful for PARACEST agents. Therefore, designing coordinatively saturated nine-coordinate complexes that undergo dissociative exchange is a useful starting point for the development of PARACEST agents. Moreover, stabilizing the nine-coordinate ground state, for example through hydrogen-bond interactions, would lead to a larger energy gap between the ground and transition states, potentially leading to slow water-exchange rates. Stabilization of the nine-coordinate ground state leads to slow water-exchange rates, which at first glance seems opposite to the fast water-exchange rate observed for complex **3** when the nine-coordinate transition state was stabilized. However, the opposite trends in water-exchange rates can be rationalized based on the difference in the mechanism of exchange: *dissociative* mechanisms have slow water-exchange rates with stable nine-coordinate *ground states*, and *associative* mechanisms have fast water-exchange rates with stable nine-coordinate *transition states*. 

### 2.2. Modification of the Charge of Ln^III^-Containing Complex

**Figure 4:**
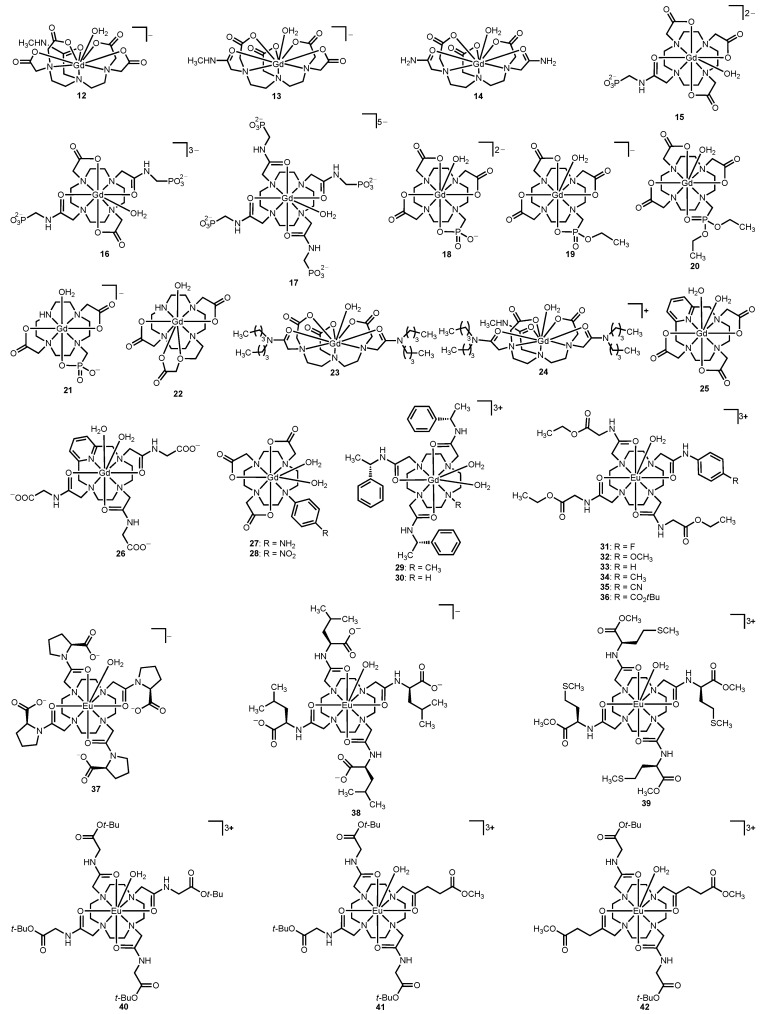
Representative Gd^III^- and Eu^III^-containing complexes that relate the influence of charge (compounds **12**–**26**) and electron density of coordinating atoms (compounds **27**–**42**) to water-exchange rate [38,39,40,41,42,43,44,45,46,47,48,49].

The charge of Ln^III^-containing complexes is another important determinant of the magnitude of water-exchange rate, and this section describes the influence of complex charge and electron density of the coordinating atoms on water-exchange rate using complexes **12**–**42** as examples (Figure 4 and Table 2). It is important to note that modification of charge toward positively charged complexes is expected to lead to increased proton-exchange rates [11]; however, discussion of proton-exchange rates is beyond the scope of this review. Also, the differences in water-exchange rates described in this section are explained in terms of charge density only, although the charge density at the Ln^III^ center closely correlates with Lewis acidity of the Ln^III^ ion. The influence of the charge of a complex on water-exchange rates is exemplified by the 2- to 8-times slower water-exchange rate that is observed for monoamide complexes **12** and **13** and bisamide complex **14** compared to the all carboxylate complex **2** [38,39]. Not only is the charge of a Ln^III^-containing complex important for tuning water-exchange rates, but the charge density at the Ln^III^ center is equally important. The atoms directly coordinating to a Ln^III^ ion tend to have the greatest impact on the charge density at the Ln^III^ center. Modifying the coordinating atoms can change the charge density, thereby altering the water-exchange rate. For example, a slowing of water-exchange rates is observed in macrocyclic amide derivatives **15**–**17** relative to the all-carboxylate macrocyclic complex **1** [40]. This slowing of water-exchange rates occurs due to the higher positive charge density at the Gd^III^ center, regardless of the increasing negative charge of the phosphonate side chains in the series **15**–**17**. The slowing of water-exchange rates in amide-containing complexes is proportional to the number of amide groups that coordinate to the Ln^III^ ion.

**Table 2:** Water-exchange rates of complexes **12**–**42** discussed in section 2.2, determined from ^17^O-NMR spectroscopy and CEST spectra for Gd^III^- and Eu^III^-containing complexes, respectively.

Complex	*k_ex_* (×10^6^ s^−1^)	Ln^III^ ion	Reference
**12**	1.9	Gd^III^	[39]
**13**	1.3	Gd^III^	[39]
**14**	0.85 (37 °C)	Gd^III^	[38]
**15**	0.77	Gd^III^	[40]
**16**	0.16	Gd^III^	[40]
**17**	0.038	Gd^III^	[40]
**18**	80	Gd^III^	[41]
**19**	20	Gd^III^	[41]
**20**	4.4	Gd^III^	[41]
**21**	78.7	Gd^III^	[42]
**22**	0.457	Gd^III^	[42]
**23**	0.98	Gd^III^	[43]
**24**	0.60	Gd^III^	[43]
**25**	14.1	Gd^III^	[44]
**26**	6.29	Gd^III^	[44]
**27**	17.6	Gd^III^	[45]
**28**	7.4	Gd^III^	[45]
**29**	1.7	Gd^III^	[46]
**30**	0.66	Gd^III^	[46]
**31**	0.0069	Eu^III^	[47]
**32**	0.0051	Eu^III^	[47]
**33**	0.0037	Eu^III^	[47]
**34**	0.0033	Eu^III^	[47]
**35**	0.0031	Eu^III^	[47]
**36**	0.0028	Eu^III^	[47]
**37**	0.018	Eu^III^	[48]
**38**	0.012	Eu^III^	[48]
**39**	0.005	Eu^III^	[48]
**40**	0.00290	Eu^III^	[49]
**41**	0.00253	Eu^III^	[49]
**42**	0.00211	Eu^III^	[49]

In general, faster water-exchange rates are observed in complexes with less positive charges. For example, an 18- and 4.5-fold difference in water-exchange rate was observed between negatively charged complexes **18** and **19** and structurally similar, but neutral complex **20** [41]. Another example demonstrated that the water-exchange rate of negatively charged Gd^III^-containing 1,4,7,10-tetraazacyclododecane-1,4,7-triacetate (DO3A) derivative **21** with a monodentate phosphonate group displayed a water-exchange rate that is 170-times faster than that of the neutral DO3A derivative **22** with a bidentate ethoxyacetate moiety [42]. The difference in water-exchange rate between negatively charged and neutral complexes is possibly due to the difference in charge between the two complexes but is likely also due to the change in the functional groups (discussed in Section 2.3, Section 2.4). Based on the correlation between fast water-exchange rates and positive charges, complexes with less positive charge at the Gd^III^ center are desirable for designing Gd^III^-containing *T*_1_-shortening agents with fast water-exchange rates.

On the other hand, reducing the water-exchange rate is desirable when designing PARACEST agents, and complexes with high positive charges on the Ln^III^ ion tend to lead to slow water-exchange rates. As mentioned earlier, the observed 8.2-fold difference in water-exchange rate between negatively charged linear complex **2** and the analogous neutral linear complex **14** at 37 °C is an example of slowing of water-exchange rates with higher overall positive charge and higher positive charge on the Ln^III^ ion [38]. Similarly, a 1.6-fold difference in water-exchange rate was observed between neutral, linear complex **23** and the analogous positively charged complex **24** [43]. Complexes **23** and **24** are structurally similar but have different charges; consequently, the slowing of water-exchange rate can be attributed at least partially to the difference in charge between **23** and **24**. In another example, a 2.2-fold difference in water-exchange rate was observed between neutral, macrocyclic Gd^III^-containing complex **25** and neutral glycine derivative **26** [44]. Although complexes **25** and **26** are both neutral, the charge distribution on the complexes is different. Complex **26** has the negative ligand-based charges farther away from the Gd^III^ ion than in complex **25**, resulting in a lower density of positive charge close to the Gd^III^ ion in complex **25** relative to **26**. This difference in charge density at the Gd^III^ center likely is responsible for the differences in water-exchange rates between complexes **25** and **26**. Observations that slow water-exchange rates occur with more positive charges suggest that complexes with more positive charges are potentially useful in designing PARACEST agents with slow water-exchange rates.

To further probe the influence of charge density near a Ln^III^ ion on water-exchange rates, the electronic effects of coordinating atoms have been studied. As an example, Aime and co-workers demonstrated that the water-exchange rates of neutral DO3A-type complexes can be varied by altering the electronic properties of coordinating macrocyclic nitrogen atoms [45]. In this example, complexes **27** and **28** differ in the substituent on the *para*-position of the phenyl group attached to a coordinating macrocyclic nitrogen atom. A 2.4-fold faster water-exchange rate was observed with complex **27**, with an electron-donating amino group, relative to complex **28**, with an electron-withdrawing nitro group [45]. Moreover, the influence of electron-donating methyl groups on water-exchange rates of triamide derivatives of DO3A-type complexes has also been investigated. Complex **29** that contains an electron-donating methyl substituent displayed a 2.6-fold faster water-exchange rate than non-methyl-containing analog **30** [46]. The relatively fast water-exchange rates in complexes **27** and **29** compared to **28** and **30** can be attributed to the electron density on the macrocyclic nitrogen atoms. This density can neutralize some of the positive charge on the Ln^III^ ion resulting in weak interactions with water and facilitate the dissociation of coordinated water from the Ln^III^ ion. In addition to electron density, steric hindrance at the water-coordination site caused by the methyl group in complex **29** (described in Section 2.3) is likely another contributing factor to the faster water-exchange rate of complex **29** relative to complex **30**. 

The influence of the electron density of macrocyclic nitrogen donors on water-exchange rates is similar to the influence of electron density on arm donors. In one study, Sherry and co-workers investigated the series of complexes **31**–**36** containing substituents with different electronic properties, and demonstrated that water-exchange rate can be tuned by varying the electron density on the coordinating amide group. In this study, mesomeric electron-donating groups (OCH_3_) led to fast exchange rates, and mesomeric electron-withdrawing groups (CO_2_*t-*Bu and CN) led to slow exchange. However, the opposite trend was reported for inductively donating (CH_3_) and withdrawing (F) groups [47]. In another example, a series of three complexes (compounds **37**–**39**) was investigated, where complexes **37** and **38** displayed 3.6- and 2.4-times faster water-exchange rates, respectively, relative to complex **39**. This observation was expected based on the calculated Mulliken charges on the coordinating carbonyl oxygen atoms. In this series of complexes, the negative charge on the coordinating carbonyl oxygen atoms ranges from most negative in complex **37** to least negative in complex **39** [48]. The idea that lower charge density on the coordinating atom leads to slower water-exchange rates was expanded to investigate the suitability of ketones to achieve slow water-exchange rates desirable for PARACEST agents [49]. In this study of complexes **40**–**42**, water-exchange rates were 1.1- to 1.4-times faster in complex **40** relative to complexes **41** and **42**, respectively. These observations are consistent with water-exchange rate being dependent on the number of poorly electron donating ketone-donor arms in the complex. 

Based on the studies described in this section, negatively charged complexes with carboxylate and phosphonate donors lead to fast water-exchange rates compared to positively charged complexes with amide donors. Moreover, water-exchange rates become faster as a function of the negative charge of the complex, low positive charge density at the Ln^III^ center, or both. These features that lead to fast water-exchange rates are desirable for the design of *T*_1_-shortening agents. The opposite is true for PARACEST agents, where positively charged complexes with amide and ketone donors tend to be useful as PARACEST agents because complexes with high positive charges favor slow water-exchange rates. However, complexes with poorly donating amide and ketone groups tend to display lower stability relative to negatively charged complexes with strong carboxylate donors, and stability of these complexes is critical in designing contrast agents. 

### 2.3. Modification of Steric Hindrance at the Site of Water Coordination

**Figure 5:**
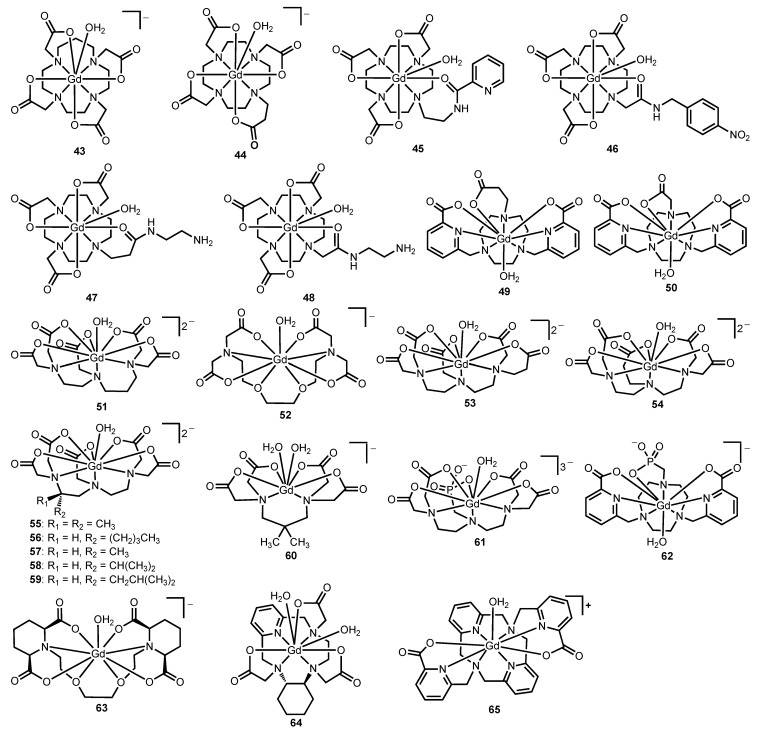
Representative Gd^III^-containing complexes **43**–**65** that relate the influence of steric hindrance at the water-coordination site to water-exchange rate [10,50,51,52,53,54,55,56,57,58,59,60,61,62].

Another parameter that influences water-exchange rates is the degree of steric hindrance at the water-coordination site. This parameter is related to mechanism of exchange and isomer ratio (described in Section 2.5); increased steric hindrance leads to faster water-exchange rates in complexes that undergo dissociative water exchange because crowding the water-coordination site favors dissociation of the coordinated water molecule, which is the rate-limiting step. The influence of steric hindrance at the site of water coordination on water-exchange rate is described in this section using complexes **43**–**65** (Figure 5 and Table 3).

**Figure 6:**
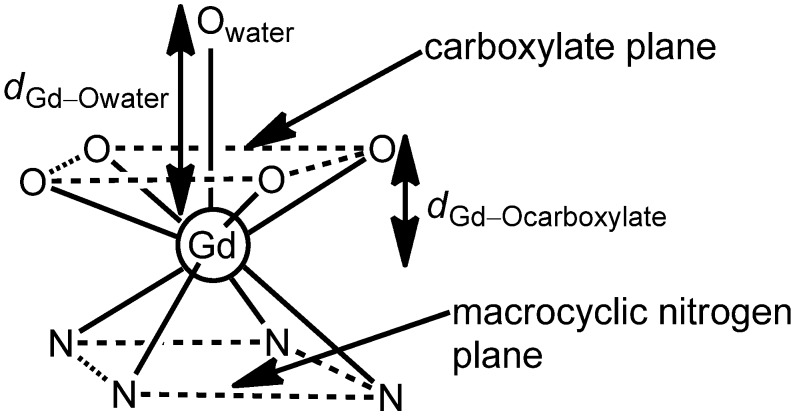
Coordination polyhedron of Gd^III^-containing complex **43** [10].

Merbach and coworkers demonstrated that complex **43**, an analog of macrocyclic complex **1** with an extended macrocyclic backbone that is one carbon longer than the macrocyclic backbone of complex **1**, has a 66-fold faster water-exchange rate relative to complex **1**. The difference in water-exchange rates was attributed to the increased steric encumbrance at the site of water coordination that results from the difference between the five-membered and six-membered rings formed between the macrocycles and the Ln^III^ ions [10]. The argument of increased steric hindrance is supported by the differences in the Gd^III^–O_carboxylate_ distances and the O_carboxylate_–Gd^III^–O_carboxylate_ angles (Figure 6). Although complexes **1** and **43** have similar Gd^III^–O_water_ distances of 2.45 and 2.48 Å, respectively, they have markedly different Gd^III^–O_carboxylate_ distances of 0.70 and 0.83 Å, respectively. These data indicate that the negatively charged carboxylate plane is closer to the axially coordinated water molecule in complex **43** than in complex **1**. This argument is further supported by the differences in the O_carboxylate_–Gd^III^–O_carboxylate_ angles (136.7 and 142.7° for complex **43** vs 146° for **1**) implying that the carboxylate plane around the Gd^III^ ion is more compact in complex **43** compared to complex **1** [10]. 

The steric difference between five and six-membered rings in chelation also can be observed in the arms of the complexes. Complex **44**, with an extended carboxylate arm that is one carbon longer than the arms in complex **1**, displayed a 15-fold faster water-exchange rate than complex **1**. This difference is likely due to the increased steric hindrance at the site of water coordination, similar to the system with an extended macrocycle [50]. Based on the water-exchange rates of **43** and **44**, extending the macrocyclic backbone has a more pronounced effect in increasing the water-exchange rates of DOTA-type complexes compared to extending one of the arms.

**Table 3:** Water-exchange rates of Gd^III^-containing complexes **43**–**65** discussed in section 2.3 from ^17^O-NMR spectroscopy.

Complex	*k_ex_* (×10^6^ s^−1^)	Reference
**43**	270	[10]
**44**	61	[50]
**45**	110	[51]
**46**	1.6	[52]
**47**	81.2	[53]
**48**	1.1	[53]
**49**	86	[54]
**50**	0.6	[54]
**51**	330	[55]
**52**	31	[56]
**53**	80	[50]
**54**	31	[50]
**55**	18 (37 °C)	[57]
**56**	12 (37 °C)	[57]
**57**	11 (37 °C)	[57]
**58**	10 (37 °C)	[57]
**59**	9.26 (37 °C)	[57]
**60**	43	[58]
**61**	11	[59]
**62**	34	[54]
**63**	59	[60]
**64**	29	[61]
**65**	63	[62]

In another example, DOTA-monoamide derivative **45**, with an amide group separated by an ethylene bridge from the macrocyclic nitrogen, displayed a water-exchange rate that is 69-times faster than the analogous DOTA-monoamide complex **46** [51,52]. The fast water-exchange rate of **45** is likely due to the steric hindrance at the water-coordination site, imposed by the seven membered chelate formed by the ethylene bridge between the macrocyclic nitrogen and the amide oxygen compared to the five membered chelate in DOTA-monoamide complex **46**. A similar trend in water-exchange rates was observed between DOTA-monoamide complexes **47** and **48** where complex **47** displayed a water-exchange rate that is 74-times faster than that of complex **48** [53]. The faster water-exchange rate of complex **47** relative to complex **48** is possibly due to the steric constraints at the site of water-coordination caused by the six membered chelate in **47** relative to the five membered chelate in **48**. Similar to the fast water-exchange rates observed for DOTA-monoamide derivatives **45** and **47**, a 143-fold difference in water-exchange rate was observed between triaza-macrocyclic complex **49** with a propionate arm and the analogous triaza-macrocyclic complex **50** with an acetate arm. The difference in water-exchange rate between complexes **49** and **50** was reportedly due to the increased steric hindrance at the site of water coordination caused by the extended propionate arm [54]. These studies suggest that the steric environment caused by ring size is generalizable to different sizes of macrocyclic ligand backbones.

The work on elongation of the macrocyclic backbones was extended into linear diethylene triamine pentaacetate (DTPA)-type systems by Merbach and coworkers. In one study, complex **51**, which is structurally similar to complex **2** but with an extra carbon in the nitrogen backbone, displayed a 100-fold faster water-exchange rate relative to that of complex **2**. The faster water-exchange rate of **51** relative to **2** was reported to be due to the steric hindrance caused by the extra carbon in the backbone of **51** [55]. In another example, complex **52** with an ether-based oxy-ethylene bridge in the backbone led to a water-exchange rate that is 9.4-times faster than that of complex **2**. The difference in water-exchange rate between **2** and **52** can be attributed to the 3% longer Gd–O_water_ distance in **52** compared to **2**, possibly due to steric hindrance caused by the ethylene bridge on the backbone of **52**, which facilitates the dissociation of the coordinated water molecule [56]. However, because there are multiple structural differences between complexes **2** and **52**, it is likely that no single difference is responsible for the entire change in water-exchange rate. 

In addition to extending the linear backbone, DTPA-type complexes with extended arms have been synthesized to study the influence of arm extensions on water-exchange rates. For example, a 24-fold difference in water-exchange rate was observed between DTPA analog **53** with an extended carboxylate arm and the parent complex **2** [50]. Furthermore, with DTPA-type complexes, the position of the extended carboxylate arm (whether terminal or central) also influences the water-exchange rate. Complex **53**, with a terminal extended carboxylate arm, has a water-exchange rate that is 2.6-times faster than that of complex **54** with a central extended carboxylate arm [50]. This observation implies that the extension of terminal carboxylates causes more steric hindrance at the site of water coordination and leads to faster water-exchange rates compared to extension of the central carboxylate arm. Unlike with macrocyclic complexes, extension of the nitrogen backbone with linear complexes generally has a higher impact on water-exchange rates than extending one of the carboxylate arms.

In addition to extending multidentate ligand backbones and arms, the influence of steric crowding caused by backbone substitution on the water-exchange rates was studied using the series of linear complexes **55**–**59** [57]. In this series, the complexes differ from one another in the alkyl-group substitution on the ethylene carbons. The dialkyl substituted complex **55** has a water-exchange rate that is 2.6-times faster than complex **2** at 37 °C, and monoalkyl substituted complexes **56**–**59** display water-exchange rates that are 1.3- to 1.7-times faster than the parent unsubstituted complex **2** at 37 °C. The faster water-exchange rates of the alkyl substituted complexes relative to parent complex **2** are likely due to the increased steric hindrance in the inner-coordination sphere [57]. A similar study demonstrated that dimethyl-substituted complex **60** had a 1.3-fold faster water-exchange rate than parent complex **6** [58]. A confounding point with complex **6** is that it has two different reported water-exchange rates (102 × 10^6^ s^−1^ [33] and 33 × 10^6^ s^−1^ [58]), and the 1.3-fold difference in rates is not observed if the wrong value is used when comparing complexes **6** and **60**. As with the other systems, this result is likely due to the increased steric hindrance from the methyl substitution. 

In addition to using alkyl groups to investigate the influence of steric crowding on water-exchange rates, complexes containing bulky phosphonate arms display faster water-exchange rates relative to complexes with no phosphonates. This difference in rates is likely at least partially due to the steric hindrance at the site of water-coordination caused by the large size of the phosphonate groups. For example, phosphonate-containing complex **61** displayed a water-exchange rate that is 3.5-times faster than that of the parent complex **2** [59]. In another example, phosphonate-containing triaza-macrocyclic complex **62** displayed a 57-fold faster water-exchange rate relative to the non-phosphonate-containing analog **50** [54]. 

Rigidifying the backbone of polyaminopolycarboxylate-based ligands is another strategy that often results in faster water-exchange rates, possibly due to enhanced steric crowding caused by substituents used to rigidify the ligand backbone. For example, backbone rigidification in complex **63** using two piperidine moieties led to a 1.9-fold faster water-exchange rate than non-rigid complex **52** [60]. In another example, complex **64** with a cyclohexylene-bridge-containing rigidified macrocycle led to a water-exchange rate that was 2.4-fold faster than that of non-rigid **25** [61]. Another example demonstrated that a rigid macrocyclic ligand framework based on diazapyridinophane (complex **65**) displayed a 15-fold faster water-exchange rate than complex **1** [62]. The faster water-exchange rates of rigidified complexes **63**–**65** with respect to non-rigid analogs **52**, **25**, and **1** are likely due to a variety of differences, but one of the influences is the increased steric hindrance brought about by the cyclic functionalities that enhance the rigidity of the backbone.

Increasing steric hindrance at the site of water coordination—using strategies including extension of ligand backbone or arms, incorporating bulky phosphonates, introducing bulky alkyl groups on the ligand backbone, and rigidification of the ligand backbone—leads to complexes with fast water-exchange rates; therefore, these strategies are desirable in *T*_1_-shortening agents. However, it is important to note that extension of ligand backbone and arms often leads to less stable complexes relative to analogous complexes without backbone and arm extensions, and stability is a critical consideration in the design of contrast agents. On the other hand, releasing steric encumbrance at the site of water coordination using less sterically demanding coordinating groups leads to complexes with slow water-exchange rates that might be desirable for use as PARACEST agents.

### 2.4. Modification of the Ligand Side Chains

**Figure 7:**
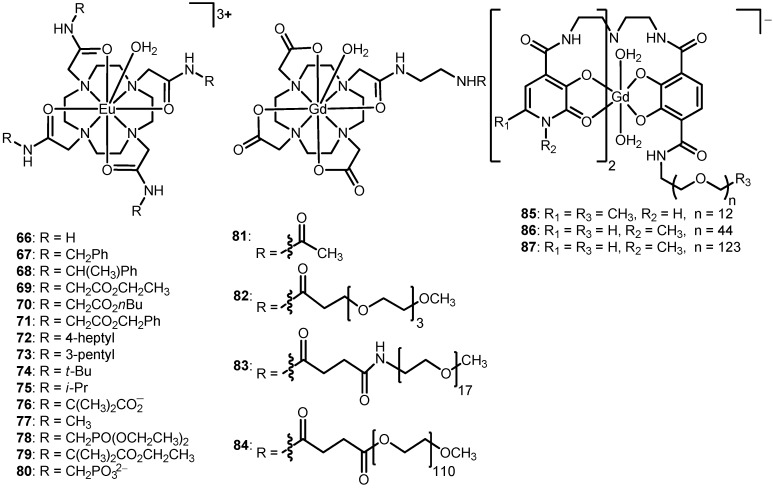
Representative complexes **66**–**87** that relate the influence of bulkiness and polarity of ligand side chains and water-exchange rates [15,27,63,64,65,66,67,68,69].

The water-exchange rates of Ln^III^-containing complexes tend to depend on the chemical nature of ligand side chains including bulkiness, polarity, and charge as has been described to some extent in the previous sections. This section focuses on the influence of ligand side chains on water-exchange rate using complexes **4** (Figure 2), **40** (Figure 4) and **66**–**87** (Figure 7 and Table 4, Table 5). For example, a series of Eu^III^-tetraamide-based complexes (**40** and **66**–**71**) was synthesized to investigate the influence of ligand side chains in terms of water-accessible surface area (calculated from molecular modeling) on water-exchange rates [63]. The complexes in the series likely undergo dissociative water exchange based on their nine coordinate ground state structures. Based on the findings of this study, the water-exchange rate decreased with decreasing water-accessible surface area (**66** > **40** > **67** > **68** > **69** > **70** = **71**) [63]. The rationale used to explain these data is that large water-accessible surfaces give rise to a large amount of second-sphere hydration, subsequently decreasing the activation energy for the water-exchange process. Moreover, any substituent that facilitates the access of incoming water molecules through second-sphere hydration was reported to lead to fast water-exchange rates. On the other hand, substituents on the ligand side chains that hinder the access of incoming water molecules lead to slow water-exchange rates [63].

**Table 4:** Water-exchange rates of Eu^III^-containing DOTA-tetraamide complexes **40** and **66**–**80** discussed in Section 2.4.

Complex	*k_ex_* (×10^6^ s^−1^)	Side chain	Method	Reference
**40**	0.007	CH_2_CO_2_*t*-Bu	NMR spectroscopy*^c^*	[63]
**66**	0.01	H	NMR spectroscopy*^c^*	[63]
**66**	0.0083*^a^*	H	^1^H-NMR spectroscopy	[65]
**67**	0.005	CH_2_Ph	NMR spectroscopy*^c^*	[63]
**68**	0.004	CH(CH_3_)Ph	NMR spectroscopy*^c^*	[63]
**69**	0.003	CH_2_CO_2_CH_2_CH_3_	NMR spectroscopy*^c^*	[63]
**69**	0.0013*^a^*	CH_2_CO_2_CH_2_CH_3_	^1^H-NMR spectroscopy	[27]
**70**	0.002	CH_2_CO_2_*n*Bu	NMR spectroscopy*^c^*	[63]
**71**	0.002	CH_2_CO_2_CH_2_Ph	NMR spectroscopy*^c^*	[63]
**72**	>1 *^a^*	4-heptyl	CEST	[15]
**73**	0.059 *^a^*	3-pentyl	CEST	[15]
**74**	0.10 *^a^*	*t*-Bu	CEST	[15]
**75**	0.027 *^a^*	*i*-Pr	CEST	[15]
**76**	0.0096 *^a^*	C(CH_3_)_2_CO_2_^−^	CEST	[15]
**77**	0.0064 *^a^*	CH_3_	^1^H-NMR spectroscopy	[65]
**78**	0.00077 *^a^*	CH_2_PO(OCH_2_CH_3_)_2_	^1^H-NMR spectroscopy	[66]
**79**	0.0048 *^a^*	C(CH_3_)_2_CO_2_CH_2_CH_3_	CEST	[15]
**80**	0.015 *^b^*	CH_2_PO_3_^2−^	^1^H-NMR spectroscopy	[27]

*^a^* for the square-antiprism (SAP) isomer in CH_3_CN, *^b^* in water, *^c^* the authors did not specify ^1^H or ^17^O.

**Table 5:** Water-exchange rates and water-coordination numbers of Gd^III^-containing polyaminopolycarboxylate-type (**81**–**84**) and HOPO-based (**4** and **85**–**87**) PEG conjugates discussed in section 2.4.

Complex	*k_ex_* (×10^6^ s^−1^)	*q*	Reference
**4**	130	2	[30]
**81**	2.7	0.9	[67]
**82**	1.5	0.9	[67]
**83**	0.83	1.0	[67]
**84**	0.67	0.8	[67]
**85**	77	1	[68]
**86**	53	1	[69]
**87**	32	1	[69]

In addition to studying the influence of water-accessible surface area on water-exchange rates, a recent study correlated the steric bulk of ligand side chains and water-exchange rates using complexes **72**–**75**. Based on this study, large amounts of steric bulk on ligand side chains resulted in fast water-exchange rates [15]. However, this trend cannot be generalized to any bulky group because there are other factors involved including polarity of the side chains. The relationship between steric bulk of ligand side chains and water-exchange rate is similar to that observed in section 2.3, where water-exchange rate increased with steric hindrance imposed at the site of water-coordination. Furthermore, at least a 17-times faster water-exchange rate was observed for more bulky heptyl-containing complex **72** relative to less bulky pentyl-containing complex **73**, and a 3.7-fold faster water-exchange rate was observed for complex **74** (with more bulky *tert*-butyl substituents) relative to **75** (with less bulky isopropyl substituents) [15]. To explain these observations, it was suggested that side chains with bulky substituents interact with one another through steric interactions making the coordinated water exposed to bulk water [15]. The exposure of coordinated water to bulk water facilitates water exchange leading to fast water-exchange rates.

Because the steric bulk and polarity of side chains are intertwined, complexes **74** and **76** were used to isolate the effects of steric bulk and polarity. Despite being similar in terms of steric bulk, complexes **74** and **76** display different water-exchange rates (Table 4), suggesting that the polarity of the side chains influences the magnitude of water-exchange rates. Complex **74** displayed a 10-fold faster water-exchange rate than complex **76** with polar carboxylate groups. This difference in water-exchange rate can be attributed to the ability of polar groups to sustain second-sphere hydration via hydrogen-bond interactions, thereby stabilizing the nine-coordinate ground state leading to slow water-exchange rates [15]. This trend in water-exchange rates is the opposite of what was reported for complexes **40** and **66**–**71** likely because both polarity and bulkiness of ligand side chains contribute to water-exchange rates and the difficulty of separating one from the other.

In general, water-exchange rates of Eu^III^-containing DOTA-tetraamide-based complexes tend to be slower when the polarity of ligand side chains is greater (primary amides < alkyl substituents < carboxylates < phosphonates) [64]. An example of this trend can be observed in the differences in water-exchange rates among complexes **66**, **69**, **77**, and **78** [primary amide-containing complex **66** > alkyl substituent-containing complex **77** > carboxylate-ester-containing complex **69** > phosphonate-ester-containing complex **78**] (Table 4) [27,65,66]. The slowing of water-exchange rates as a function of polarity can be attributed to the stabilization of the nine-coordinate ground state through hydrogen-bond interactions. Although polar ligand side chains are expected to result in slow water-exchange rates, it was observed that complexes bearing negatively charged side chains display faster water-exchange rates than their neutral analogs [64]. For example, a 2-fold difference in water-exchange rate was observed between negatively charged carboxylate-side-chain-containing analog **76** and neutral ethyl-ester-side-chain-containing complex **79** [15]. In another example, a 52-fold difference in water-exchange rate was observed between neutral phosphate-ester-side-chain-containing complex **78** and negatively charged phosphonate-side-chain-containing analog **80** [27,66]. The slower water-exchange rates of neutral ester-containing complexes relative to their negatively charged analogs is likely due to the ability of ethyl groups to block incoming water molecules, subsequently lowering the number of water molecules available for exchange [15]. The observation of complexes with negatively charged side chains leading to fast water-exchange rates is consistent with section 2.2 that relates negatively charged complexes to fast water-exchange rates.

Based on the idea of polar side chains leading to slow water-exchange rates, a recent study explored the ability of polar polyethylene glycol (PEG) to slow water-exchange rates as a function of length of PEG [67]. A series of polyaminopolycarboxylate-type complexes, **81**–**84**, were used in this study. A 4-fold difference in water-exchange rate was observed between complex **81** (without PEG) and **84** (with a PEG length of 110 monomer units) (Table 5) [67]. The difference in water-exchange rates as a function of length of PEG is likely due to the formation of a hydrogen-bond network that stabilizes the nine-coordinate ground state, and longer PEG lengths would enable more hydrogen bonding. It is important to note that the influence of steric blocking caused by PEG was minimal based on the similar water-coordination numbers (*q*) obtained for complexes **81**–**84** (Table 5) [67]. The slowing of water-exchange rates as a function of PEG length in the polyaminopolycarboxylate-type system is similar to observations in studies with HOPO-based PEG conjugates **85**–**87** [68,69]. Moreover, a 4-fold difference in water-exchange rate was observed between parent HOPO complex **4** without PEG and HOPO complex **87** with the PEG moiety with 123 monomer units (Table 5) [68,69]. The observed difference in water-exchange rate is likely due to hydrogen-bonding and steric interactions brought about by PEG moieties. 

Based on the studies described in this section, Ln^III^-containing complexes with bulky, hydrophobic side chains are desirable in designing *T*_1_-shortening agents, because they are expected to lead to fast water-exchange rates. On the other hand, Ln^III^-containing complexes with neutral hydrophilic side chains are suitable for developing PARACEST agents because hydrophilic side chains tend to result in slow water-exchange rates. 

### 2.5. Modification of TSAP/SAP Ratio for DOTA-Type Complexes

**Figure 8:**
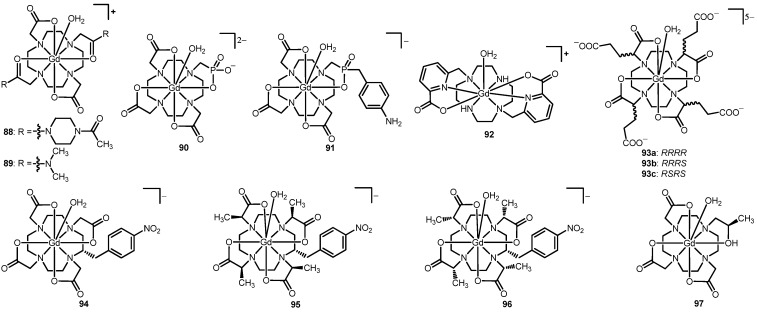
Representative complexes **88**–**97** that relate the influence of TSAP/SAP ratios with water-exchange rates of DOTA-type complexes [41,71,72,73,74,75,76,77,78].

**Figure 9:**
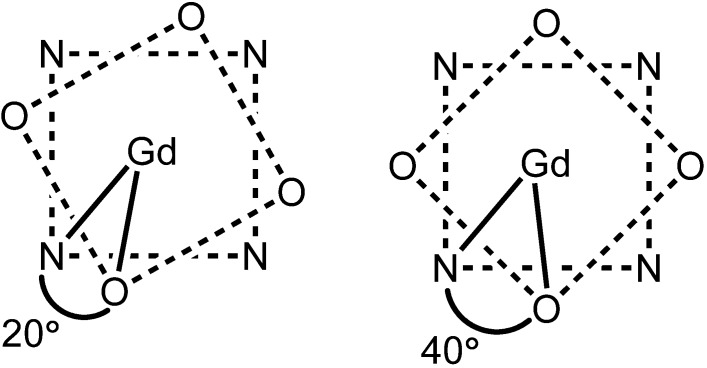
Top-down view of (left) TSAP and (right) SAP isomers with the O–Gd^III^–N twist angles indicated. In these views, the oxygen plane is above the Gd^III^ ion and the nitrogen plane is below the Gd^III^ ion.

The water-exchange rates of DOTA-type Ln^III^-containing complexes are also governed by the ratio of the twisted-square-antiprism (TSAP) to square-antiprism (SAP) isomers. In this section, the influence of the TSAP/SAP ratio on water-exchange rate will be discussed using complexes **18**–**20** and **88**–**97** (Figure 4, Figure 8 and Table 6). DOTA-type complexes exist as two diasteriomers in aqueous solution, namely TSAP and SAP that differ in the arrangement of their carboxylate arms and macrocyclic rings. Specifically, these two isomers differ in the twist angle between macrocyclic nitrogen plane and carboxylate oxygen plane: the TSAP isomer has a narrow O–Gd^III^–N twist angle (20°), and the SAP isomer has a wide O–Gd^III^–N twist angle (40°) (Figure 9). These isomers have the ability to interconvert by ring inversion or arm rotation [70].

**Table 6:** Water-exchange rates and TSAP/SAP ratios of Gd^III^-containing complexes (**18**–**20** and **88**–**97**) discussed in Section 2.5.

Complex	*k_ex_* (×10^6^ s^−1^)	TSAP/SAP ratio	*k_ex_* (×10^6^ s^−1^) for TSAP	*k_ex_* (×10^6^ s^−1^) for SAP	References
**18**	80	1.3	nr	nr	[41]
**19**	20	0.56	nr	nr	[41]
**20**	4.4	0.39	nr	nr	[41]
**88**	140 *^a^*	0.78 *^b^*	330	0.43	[71]
**89**	31 *^a^*	0.77 *^b^*	70	0.74	[71]
**90**	71	1.5	nr	nr	[72]
**91**	61.7	1.5 *^b^*	nr	nr	[73]
**92**	58	¥	nr	nr	[74]
**93a**	15.4	2	nr	nr	[75]
**93b**	9.0	0.75	nr	nr	[75]
**93c**	3.45	0.20	nr	nr	[75]
**94**	5	0.08	nr	nr	[76]
**95**	67	¥	nr	nr	[77]
**96**	8.3	0	nr	nr	[77]
**97**	4.5 (37 °C)	0.4	110	1.6	[78,79]

nr = not reported, *^a^* calculated from weighted average, *^b^* TSAP/SAP ratios obtained from Eu^III^-containing complexes.

In general, the measured water-exchange rate of DOTA-type complexes is the weighted average of the water-exchange rates of the TSAP and SAP isomers. Between the two isomers, the TSAP isomer tends to display a water-exchange rate that is approximately 50–200 times faster than the SAP isomer in DOTA-tetraamide type complexes [70,80]. The faster water-exchange rate in the TSAP isomer relative to the SAP isomer is likely due to the higher amount of steric encumbrance at the site of water coordination, which facilitates the dissociation of coordinated water similar to fast water-exchange rates (discussed in Section 2.3) due to the steric hindrance at the site of water-coordination. Because the TSAP and SAP isomers display different water-exchange rates, the water-exchange rates of DOTA-type complexes can be tuned by changing the relative abundance of the two isomers in solution using coordination-chemistry-based strategies. Complexes with sterically bulky substituents tend to favor the TSAP isomer, while complexes without bulky substituents favor the SAP isomer. However, it is important to note that relative abundance of the two isomers also depends on other factors including ionic radius, solvent, ionic strength of the solution, and salt composition [81,82,83]. 

To study the influence of steric bulk on amide nitrogen atoms on the relative abundance of the TSAP and SAP isomers, complexes **88** and **89** were investigated. The TSAP/SAP ratios of 0.78 and 0.77 were measured for Eu^III^-containing complexes **88** and **89**, respectively. These ratios are 2-times greater than Eu^III^-containing complex **1** that has a TSAP/SAP ratio of 0.36 [71]. The water-exchange rates obtained for the Gd^III^-containing complexes **88** and **89** indicated that the TSAP isomers displayed 782- and 95-times faster water-exchange rates, respectively, than the SAP isomers [71].

In addition to introducing steric bulk on amide nitrogen atoms, other bulky DOTA-type complexes increase the population of the TSAP isomer relative to the SAP isomer, leading to faster water-exchange rates. This idea can be exemplified using phosphonate-containing DOTA-type systems [41,72,73]. In one example, a TSAP/SAP ratio of approximately 1.5 was observed for phosphonate-containing complex **90** and resulted in a 17-fold faster water-exchange rate relative to that of complex **1** [72]. In another example, a TSAP/SAP ratio of 1.5 for phosphonate-containing complex **91**, which is 4-times greater than that of complex **1**, resulted in a water-exchange rate that is 15-times faster than complex **1** [73]. Interestingly, the series of phosphonate-containing complexes **18**–**20** were used to demonstrate that the TSAP/SAP ratios are larger with complexes of more negative charge. The TSAP/SAP ratios were 1.3 and 0.56 in negatively charged complexes **18** and **19**, respectively, and 0.39 in neutral complex **20** suggesting that the TSAP isomer dominates in complexes with more negative charge. The difference in the populations of the TSAP isomers was reflected in the water-exchange rates of complexes **18**–**20**: 18- and 4.5-fold differences in water-exchange rates were observed in complexes **18** and **19**, respectively, relative to complex **20** [41]. The differences in water-exchange rates of complexes **18**–**20** are likely due to the difference in the TSAP isomer population, the difference in complex charge as described in Section 2.2, or both factors. In addition to bulky phosphonate-containing complexes, complex **92** with a bulky bis-methylene picolinate platform was explored. Complex **92** displayed a water-exchange rate that is 14-times faster than that of complex **1** [74]. The faster water-exchange rate is likely because complex **92** exists exclusively as the distorted TSAP isomer [74]. 

In addition to incorporating steric bulk on the ends of the arms farthest from the macrocycle, DOTA-type complexes with substituents at the α-positions of the pendent arms increase the abundance of the TSAP isomer. As an example, Parker and co-workers observed that a Gd^III^-containing DOTA analog with propionate groups in the α-positions of the pendent arms contains the three isomers **93a**, **93b**, and **93c**. In this series, the water-exchange rate increases with increasing TSAP/SAP ratios [75]. Interconversion between TSAP and SAP isomers in complexes **93a** and **93b** was reported to occur only through ring inversion because the bulky propionate group in the α-position of the pendent arm hinders arm rotation affording TSAP/SAP ratios that are 10- and 3.8-times higher, respectively, than in complex **93c**. The low TSAP/SAP ratio in complex **93c** is likely due to interconversion between TSAP and SAP isomers through arm rotation as well as ring inversion. The differences in the TSAP/SAP ratios are likely responsible for the 4.5- and 2.6-fold faster water-exchange rates of complexes **93a** and **93b**, respectively, relative to complex **93c** [75]. In addition to blocking arm rotation, complexes containing bulky substituents on the macrocycle were synthesized with the objective of blocking ring inversion in DOTA-type complexes. A study of complex **94** with a *p*-nitrobenzyl group on the macrocycle demonstrated that TSAP to SAP interconversion through ring inversion can be blocked. However, arm rotation persisted affording a TSAP/SAP ratio of 0.08 resulting in a water-exchange rate that is only 1.2-times faster than that of complex **1** [76]. Based on the observations that arm rotation and ring inversion can be blocked by arm and ring substitutions, respectively, complexes with both arm and ring substitutions were reported to control isomer type. Furthermore, controlling the chirality at five carbons led to preference for a single isomer. For example, complexes **95** (*S*-*SSSS*) and **96** (*S*-*RRRR*) with substituents on the macrocycle as well as the pendant arms were reported to exist exclusively as TSAP and SAP isomers, respectively [77]. The water-exchange rate of **95** (*S*-*SSSS*) in the TSAP form was 8-times faster than the water-exchange rate of **96** (*S*-*RRRR*) in the SAP form [77]. These examples substantiate that complexes existing exclusively in the TSAP form have faster water-exchange rates relative to complexes existing in the SAP form. 

In addition to experimental studies carried out to tune TSAP/SAP ratios in DOTA-type complexes, a computational study was carried out on complex **97** to understand the rationale behind the TSAP isomer leading to fast water-exchange rates [78]. This computational study suggested that the TSAP isomer displays hydrogen-bond interactions between pendant arms and second-sphere water, enabling the coordinated water to exchange readily with bulk water, leading to fast water-exchange rates. On the other hand, the SAP isomer is expected to form hydrogen-bond interactions between second-sphere and inner-sphere water, stabilizing the ground state in dissociative exchange processes and leading to slow water-exchange rates. Based on this study, increasing the ability of pendant arms to form hydrogen bonds without lengthening side chains is likely to result in large proportions of the TSAP isomer with DOTA-type complexes. Although the predictions of this study at first glance appear to be in contrast with the studies discussed in section 2.4, the difference is likely because the side chains of complexes **81**–**87** are long compared to side chain of complex **97**. The length of the side chain influences how far from the Ln^III^ ion hydrogen bonding occurs, and this distance is an important difference between the complexes described in sections 2.4 and 2.5.

Complexes with large TSAP/SAP ratios are expected to lead to fast water-exchange rates necessary for conventional *T*_1_-shortening agents because the TSAP isomers display fast water-exchange rates. Large TSAP/SAP ratios are likely to form with complexes that contain bulky substituents on the α-position of pendant arms, on the macrocycle, and on amide nitrogen atoms as well as from complexes with side chains that facilitate hydrogen-bonding interactions near the inner-sphere water. On the other hand, for the design of PARACEST agents, low TSAP/SAP ratios are desirable because the SAP isomers display water-exchange rates that are 1–2 orders of magnitude slower than the TSAP isomers. Complexes with low TSAP/SAP ratios are likely with low steric strain on pendant arms and the macrocycle as well as on amide nitrogen atoms. Also, side chains that hinder hydrogen-bond interactions close to the inner-sphere water might favor the SAP isomer leading to slow water-exchange rates useful for PARACEST agents.

## 3. Conclusions

Water-exchange rate is an important molecular parameter in Ln^III^-containing complexes that governs the efficiency of *T*_1_-shortening and PARACEST contrast agents for MRI. Consequently, tuning water-exchange rates of Ln^III^-containing complexes to achieve maximum efficiencies for both types of agents has led to a great deal of research. As discussed in the text, several coordination-chemistry-based strategies have been employed to tune water-exchange rates of Ln^III^-containing complexes toward the optimum values desirable for both types of agents. It is important to re-emphasize that there is overlap among the strategies discussed in this article.

Based on the influence of different coordination-chemistry-based strategies on water-exchange rate, complexes with the following properties are expected to lead to fast water-exchange rates that are desirable for the design of *T*_1_-shortening agents: that undergo associative water exchange; that are negatively charged; with steric hindrance at the site of water coordination; with side chains containing non-polar substituents; and with large TSAP/SAP ratios for DOTA-type complexes. On the other hand, complexes that undergo dissociative exchange; that are positively charged; without bulky substituents at the site of water coordination; with polar substituents on side chains; and with predominantly SAP isomers for DOTA-type complexes are expected to lead to slow water-exchange rates that are desirable for the design of PARACEST agents. Careful consideration and application of the coordination-chemistry-based strategies described in this review have the potential to enable fine-tuning of water-exchange rates towards optimal fast or slow rates. Consequently, these strategies are expected to aid the design of both *T*_1_-shortening and PARACEST contrast agents. 

## References

[1] Hermann P., Kotek J., Kubíček V., Lukeš I. (2008). Gadolinium(III) complexes as MRI contrast agents: ligand design and properties of the complexes. Dalton Trans..

[2] Caravan P. (2006). Strategies for increasing the sensitivity of gadolinium based MRI contrast agents. Chem. Soc. Rev..

[3] Terreno E., Castelli D.D., Viale A., Aime S. (2010). Challenges for molecular magnetic resonance imaging. Chem. Rev..

[4] Laurent S., Henoumont C., Vander Elst L., Muller R.N. (2012). Synthesis and physicochemical characterization of Gd-DTPA derivatives as contrast agents for MRI. Eur. J. Inorg. Chem..

[5] Dastrù W., Longo D., Aime S. (2011). Contrast agents and mechanisms. Drug Discov. Today.

[6] Weise G., Basse-Lüsebrink T.C., Kleinschnitz C., Kampf T., Jakob P.M., Stoll G. (2011). In vivo imaging of stepwise vessel occlusion in cerebral photothrombosis of Mice by ^19^F MRI. PLoS One.

[7] Caravan P., Farrar C.T., Frullano L., Uppal R. (2009). Influence of molecular parameters and increasing magnetic field strength on relaxivity of gadolinium- and manganese-based T_1_ contrast agents. Contrast Media Mol. Imaging.

[8] Caravan P., Ellison J.J., McMurry T.J., Lauffer R.B. (1999). Gadolinium(III) chelates as MRI contrast agents: structure, dynamics, and applications. Chem. Rev..

[9] Tóth É., Helm L., Merbach A., Merbach A., Helm L., Tóth É. (2013). Relaxivity of gadolinium(III) complexes: theory and mechanism. The Chemistry of Contrast Agents in Medical Magnetic Resonance Imaging.

[10] Ruloff R., Tóth É., Scopelliti R., Tripier R., Handel H., Merbach A.E. (2002). Accelerating water exchange for Gd^III^ chelates by steric compression around the water binding site. Chem. Commun..

[11] Aime S., Barge A., Botta M., Parker D., De Sousa A.S. (1997). Prototropic *vs* whole water exchange contributions to the solvent relaxation enhancement in the aqueous solution of a cationic Gd^3+^ macrocyclic complex. J. Am. Chem. Soc..

[12] Woods M., Woessner D.E., Sherry A.D. (2006). Paramagnetic lanthanide complexes as PARACEST agents for medical imaging. Chem. Soc. Rev..

[13] Viswanathan S., Kovacs Z., Green K.N., Ratnakar S.J., Sherry A.D. (2010). Alternatives to gadolinium-based metal chelates for magnetic resonance imaging. Chem. Rev..

[14] Dorazio S.J., Morrow J.R. (2012). The development of iron(II) complexes as paraCEST MRI contrast agents. Eur. J. Inorg. Chem..

[15] Mani T., Tircsó G., Togao O., Zhao P., Soesbe T.C., Takahashi M., Sherry A.D. (2009). Modulation of water exchange in Eu(III) DOTA-tetraamide complexes: considerations for *in vivo* imaging of PARACEST agents. Contrast Media Mol. Imaging.

[16] Terreno E., Castelli D.D., Cravotto G., Milone L., Aime S. (2004). Ln(III)-DOTAMGly complexes: a versatile series to assess the determinants of the efficacy of paramagnetic chemical exchange saturation transfer agents for magnetic resonance imaging applications. Invest. Radiol..

[17] Opina A.C.L., Wu Y., Zhao P., Kiefer G., Sherry A.D. (2011). The pH sensitivity of –NH exchange in LnDOTA-tetraamide complexes varies with amide substituent. Contrast Media Mol. Imaging.

[18] Powell D.H., Favre M., Graeppi N., Dhubhghaill O.M.N., Pubanz D., Merbach A.E. (1995). Solution kinetic behavior of lanthanide (III) polyaminocarboxylates from oxygen-17 NMR studies. J. Alloys Compd..

[19] Aime S., Botta M., Fasano M., Crich S.G., Terreno E. (1999). ^1^H and ^17^O-NMR relaxometric investigations of paramagnetic contrast agents for MRI. Clues for higher relaxivities. Coord. Chem. Rev..

[20] Helm L., Nicolle G.M., Merbach A.E. (2005). Water and proton exchange processes on metal ions. Adv. Inorg. Chem..

[21] Aime S., Botta M., Fasano M., Terreno E. (1999). Prototropic and water-exchange processes in aqueous solutions of Gd(III) chelates. Acc. Chem. Res..

[22] Sherry A.D., Wu Y. (2013). The importance of water exchange rates in the design of responsive agents for MRI. Curr. Opin. Chem. Biol..

[23] Helm L., Merbach A.E. (2005). Inorganic and bioinorganic solvent exchange mechanisms. Chem. Rev..

[24] Cossy C., Helm L., Merbach A.E. (1988). Oxygen-17 nuclear magnetic resonance kinetic study of water exchange on the lanthanide(III) aqua ions. Inorg. Chem..

[25] Graeppi N., Powell D.H., Laurenczy G., Zékány L., Merbach A.E. (1995). Coordination equilibria and water exchange kinetics of lanthanide(III) propylenediaminetetraacetates and other magnetic resonance imaging related complexes. Inorg. Chim. Acta.

[26] Pubanz D., González G., Powell D.H., Merbach A.E. (1995). Unexpectedly large change of water exchange rate and mechanism on [Ln(DTPA-BMA)(H^2^O)] complexes along the lanthanide(III) series. Inorg. Chem..

[27] Zhang S., Sherry A.D. (2003). Physical characteristics of lanthanide complexes that act as magnetization transfer (MT) contrast agents. J. Solid State Chem..

[28] Cohen S.M., Xu J., Radkov E., Raymond K.N., Botta M., Barge A., Aime S. (2000). Synthesis and relaxation properties of mixed gadolinium hydroxypyridinonate MRI contrast agents. Inorg. Chem..

[29] Thompson M.K., Botta M., Nicolle G., Helm L., Aime S., Merbach A.E, Raymond K.N. (2003). A highly stable gadolinium complex with a fast, associative mechanism of water exchange. J. Am. Chem. Soc..

[30] Pierre V.C., Botta M., Aime S., Raymond K.N. (2006). Tuning the coordination number of hydroxypyridonate-based gadolinium complexes: implications for MRI contrast agents. J. Am. Chem. Soc..

[31] Powell D.H., Dhubhghaill O.M.N., Pubanz D., Helm L., Lebedev Y.S., Schlaepfer W., Merbach A.E. (1996). Structural and dynamic parameters obtained from ^17^O NMR, EPR, and NMRD studies of monomeric and dimeric Gd^3+^ complexes of interest in magnetic resonance imaging: an integrated and theoretically self-consistent approach. J. Am. Chem. Soc..

[32] Aime S., Botta M., Frullano L., Crich S.G., Giovenzana G., Pagliarin R., Palmisano G., Sirtori F.R., Sisti M. (2000). GdPCP2A(H_2_O)_2_]^−^: A paramagnetic contrast agent designed for improved applications in magnetic resonance imaging. J. Med. Chem..

[33] Micskei K., Powell D.H., Helm L., Brücher E., Merbach A.E. (1993). Water exchange on gadolinium (aqua)(propylenediamine tetraacetate) complexes [Gd(H_2_O)_8_]^3+^ and [Gd(PDTA)(H_2_O)_2_]^−^ in aqueous solution: a variable-pressure, -temperature and -magnetic field ^17^O NMR study. Magn. Reson. Chem..

[34] Tirscó G., Kálmán F.K., Pál R., Bányai I., Varga T.R., Király R., Lázár I., Québatte L., Merbach A.E., Tóth É. (2012). Lanthanide complexes formed with the tri- and tetraacetate derivatives of bis(aminomethyl)phosphonic acid: equilibrium, kinetic and NMR spectroscopic studies. Eur. J. Inorg. Chem..

[35] Mato-Iglesias M., Platas-Iglesias C., Djanashvili K., Peters J.A., Tóth É., Balogh E., Muller R.N., Vander Elst L., De Blas A., Rodríguez-Blas T. (2005). The highest water exchange rate ever measured for a Gd(III) chelate. Chem. Commun..

[36] Balogh E., Mato-Iglesias M., Platas-Iglesias C., Tóth É., Djanashvili K., Peters J.A., De Blas A., Rodríguez-Blas T. (2006). Pyridine- and phosphonate-containing ligands for stable Ln complexation. Extremely fast water exchange on the Gd^III^ chelates. Inorg. Chem..

[37] Pálinkás Z., Roca-Sabio A., Mato-Iglesias M., Esteban-Gómez D., Platas-Iglesias C., de Blas A., Rodríguez-Blas T., Tóth É. (2009). Stability, Water exchange, and anion binding studies on lanthanide(III) complexes with a macrocyclic ligand based on 1,7-diaza-12-crown-4: Extremely fast water exchange on the Gd^3+^ complex. Inorg. Chem..

[38] Botteman F., Nicolle G.M., Vander Elst L., Laurent S., Merbach A.E., Muller R.N. (2002). Synthesis, variable temperature and pressure ^17^O NMR study of bis(alkylamide) derivatives of [(Gd-DTPA)(H_2_O)]^2−^–an assessment of the substitution effect on water exchange kinetics. Eur. J. Inorg. Chem..

[39] Tóth É., Burai L., Brücher E., Merbach A.E. (1997). Tuning water-exchange rates on (carboxymethyl)iminobis-(ethylenenitrilo)tetraacetate (dtpa)-type gadolinium(III) complexes. J. Chem. Soc., Dalton Trans..

[40] Zhang S., Merritt M., Woessner D.E., Lenkinski R.E., Sherry A.D. (2003). PARACEST agents: modulating MRI contrast via water proton exchange. Acc. Chem. Res..

[41] Lebdušková P., Hermann P., Helm L., Tóth É., Kotek J., Binnemans K., Rudovský J., Lukeš I., Merbach A.E. (2007). Gadolinium(III) complexes of mono- and diethyl esters of monophosphonic acid analogue of DOTA as potential MRI contrast agents: solution structures and relaxometric studies. Dalton Trans..

[42] Polasek M., Caravan P. (2013). Is macrocycle a synonym for kinetic inertness in Gd(III) complexes? Effect of coordinating and noncoordinating substituents on inertness and relaxivity of Gd(III) chelates with DO3A-like ligands. Inorg. Chem..

[43] Jászberényi Z., Tóth É., Kálai T., Király R., Burai L., Brücher E., Merbach A.E., Hideg K. (2005). Synthesis and complexation properties of DTPA-*N*,*N′′*-bis[bis(*n*-butyl)]-*N′*-methyl-tris(amide). Kinetic stability and water exchange of its Gd^3+^ complex. Dalton Trans..

[44] Rojas-Quijano F.A., Benyó E.T., Tirscó G., Kálmán F.K., Baranyai Z., Aime S., Sherry A.D., Kovács Z. (2009). Lanthanide(III) complexes of tris(amide) PCTA derivatives as potential bimodal magnetic resonance and optical imaging agents. Chem. Eur. J..

[45] Terreno E., Boniforte P., Botta M., Fedeli F., Milone L., Mortillaro A., Aime S. (2003). The water-exchange rate in neutral heptadentate DO3A-like Gd^III^ complexes: effect of the basicity at the macrocyclic nitrogen site. Eur. J. Inorg. Chem..

[46] Botta M., Aime S., Barge A., Bobba G., Dickins R.S., Parker D., Terreno E. (2003). Ternary complexes between cationic Gd^III^ chelates and anionic metabolites in aqueous solution: an NMR relaxometric study. Chem. Eur. J..

[47] Ratnakar S.J., Woods M., Lubag A.J.M., Kovács Z., Sherry A.D. (2008). Modulation of water exchange in europium(III) DOTA-tetraamide complexes via electronic substituent effects. J. Am. Chem. Soc..

[48] Viswanathan S., Ratnakar S.J., Green K.N., Kovacs Z., De León-Rodríguez L.M, Sherry A.D. (2009). Multi-frequency PARACEST agents based on europium(III)-DOTA-tetraamide ligands. Angew. Chem. Int. Ed..

[49] Green K.N., Viswanathan S., Rojas-Quijano F.A., Kovacs Z., Sherry A.D. (2011). Europium(III) DOTA-derivatives having ketone donor pendant arms display dramatically slower water exchange. Inorg. Chem..

[50] Jászberényi Z., Sour A., Tóth É., Benmelouka M., Merbach A.E. (2005). Fine-tuning water exchange on Gd^III^ poly(amino carboxylates) by modulation of steric crowding. Dalton Trans..

[51] Congreve A., Parker D., Gianolio E., Botta M. (2004). Steric control of lanthanide hydration state: fast water exchange at gadolinium in a mono-amide ‘DOTA’ complex. Dalton Trans..

[52] Tóth É., Pubanz D., Vauthey S., Helm L., Merbach A.E. (1996). The role of water exchange in attaining maximum relaxivities for dendrimeric MRI contrast agents. Chem. Eur. J..

[53] Tei L., Gugliotta G., Baranyai Z., Botta M. (2009). A new bifunctional Gd^III^ complex of enhanced efficacy for MR-molecular imaging applications. Dalton Trans..

[54] Nonat A., Giraud M., Gateau C., Fries P.H., Helm L., Mazzanti M. (2009). Gadolinium(III) complexes of 1,4,7-triazacyclononane based picolinate ligands: simultaneous optimization of water exchange kinetics and electronic relaxation. Dalton Trans..

[55] Laus S., Ruloff R., Tóth É., Merbach A.E. (2003). Gd^III^ complexes with fast water exchange and high thermodynamic stability: potential building blocks for high-relaxivity MRI contrast agents. Chem. Eur. J..

[56] Yerly F., Hardcastle K.I., Helm L., Aime S., Botta M., Merbach A.E. (2002). Molecular dynamics simulation of [Gd(egta)(H_2_O)]^−^ in aqueous solution: Internal motions of the Poly(amino carboxylate) and water ligands, and rotational correlation times. Chem. Eur. J..

[57] Laurent S., Botteman F., Vander Elst L., Muller R.N. (2004). Optimising the design of paramagnetic MRI contrast agents: influence of backbone substitution on the water exchange rate of Gd-DTPA derivatives. MAGMA.

[58] Forgács A., Giovenzana G.B., Botta M., Brücher E., Tóth I., Baranyai Z. (2012). Influence of *gem*-dimethyl substituition on the stability, kinetics and relaxometric properties of PDTA complexes. Eur. J. Inorg. Chem..

[59] Kotek J., Lebdušková P., Hermann P., Vander Elst L., Muller R.N., Geraldes C.F.G.C., Maschmeyer T., Lukeš I., Peters J.A. (2003). Lanthanide(III) complexes of novel mixed carboxylic-phosphorus acid derivatives of diethylenetriamine: A step towards more efficient MRI contrast agents. Chem. Eur. J..

[60] Tei L., Baranyai Z., Cassino C., Fekete M., Kálmán F.K., Botta M. (2012). Solution properties of the Ln^III^ complexes of a novel octadentate chelator with rigidified iminodiacetate arms. Dalton Trans..

[61] Port M., Raynal I., Vander Elst L., Muller R.N., Dioury F., Ferroud C., Guy A. (2006). Impact of rigidification on relaxometric properties of a tricyclic tetraazatriacetic gadolinium chelate. Contrast Med. Mol. Imaging.

[62] Roca-Sabio A., Bonnet C.S., Mato-Iglesias M., Esteban-Gómez D., Tóth É., de Blas A., Rodríguez-Blas T., Platas-Iglesias C. (2012). Lanthanide complexes based on a diazapyridinophane platform containing picolinate pendants. Inorg. Chem..

[63] Aime S., Barge A., Batsanov A.S., Botta M., Castelli D.D., Fedeli F., Mortillaro A., Parker D., Puschmann H. (2002). Controlling the variation of axial water exchange rates in macrocyclic lanthanide(III) complexes. Chem. Commun..

[64] Zhang S., Jiang X., Sherry A.D. (2005). Modulation of the lifetime of water bound to lanthanide metal ions in complexes with ligands derived from 1,4,7,10-Tetraazacyclododecane tetraacetate (DOTA). Helv. Chim. Acta.

[65] Aime S., Barge A., Bruce J.I., Botta M., Howard J.A.K., Moloney J.M., Parker D., de Sousa A.S., Woods M. (1999). NMR, Relaxometric, and structural studies of the hydration and exchange dynamics of cationic lanthanide complexes of macrocyclic tetraamide ligands. J. Am. Chem. Soc..

[66] Zhang S., Wu K., Sherry A.D. (2001). Gd^3+^ complexes with slowly exchanging bound-water molecules may offer advantages in the design of responsive MR agents. Invest. Radiol..

[67] Siriwardena-Mahanama B.N., Allen M.J. (2013). Modulating water-exchange rates of lanthanide(III)-containing polyaminopolycarboxylate-type complexes using polyethylene glycol. Dalton Trans..

[68] Thompson M.K., Doble D.M.J., Tso L.S., Barra S., Botta M., Aime S., Raymond K.N. (2004). Hetero-tripodal hydroxypyridonate gadolinium complexes: syntheses, relaxometric properties, water exchange dynamics, and human serum albumin binding. Inorg. Chem..

[69] Doble D.M.J., Botta M., Wang J., Aime S., Barge A., Raymond K.N. (2001). Optimization of the relaxivity of MRI contrast agents: Effect of poly(ethylene glycol) chains on the water-exchange rates of Gd^III^ complexes. J. Am. Chem. Soc..

[70] Dunand F.A., Aime S., Merbach A.E. (2000). First ^17^O-NMR observation of coordinated water on both isomers of [Eu(DOTAM)(H_2_O)]^3+^: a direct access to water exchange and its role in the isomerization. J. Am. Chem. Soc..

[71] Zhang S., Kovacs Z., Burgess S., Aime S., Terreno E., Sherry A.D. (2001). {DOTA-bis(amide)}lanthanide complexes: NMR evidence for differences in water-molecule exchange rates for coordination isomers. Chem. Eur. J..

[72] Rudovský J., Cígler P., Kotek J., Hermann P., Vojtíšek P., Lukeš I., Peters J.A., Vander Elst L., Muller R.N. (2005). Lanthanide(III) complexes of a mono(methylphosphonate) analog of H_4_dota: the influence of protonation of the phosphonate moiety on the TSAP/SAP isomer ratio and the water exchange rate. Chem. Eur. J..

[73] Rudovský J., Kotek J., Hermann P., Lukeš I., Mainero V., Aime S. (2005). Synthesis of a bifunctional monophosphinic acid DOTA analogue ligand and its lanthanide(III) complexes. A gadolinium(III) complex endowed with an optimal water exchange rate for MRI applications. Org. Biomol. Chem..

[74] Rodríguez-Rodríguez A., Esteban-Gómez D., de Blas A., Rodríguez-Blas T., Fekete M., Botta M., Tripier R., Platas-Iglesias C. (2012). Lanthanide(III) complexes with ligands derived from a cyclen framework containing pyridinecarboxylate pendants. The effect of steric hindrance on the hydration number. Inorg. Chem..

[75] Woods M., Aime S., Botta M., Howard J.A.K., Moloney J.M., Navet M., Parker D., Port M., Rousseaux O. (2000). Correlation of water exchange rate with isomeric composition in diastereomeric gadolinium complexes of tetra(carboxyethyl)dota and related macrocyclic ligands. J. Am. Chem. Soc..

[76] Woods M., Kovacs Z., Kiraly R., Brücher E., Zhang S., Sherry A.D. (2004). Solution dynamics and stability of lanthanide(III) (*S*)-2-(*p*-nitrobenzyl)DOTA complexes. Inorg. Chem..

[77] Woods M., Kovacs Z., Zhang S., Sherry A.D. (2003). Towards the rational design of magnetic resonance imaging contrast agents: isolation of the two coordination isomers of lanthanide DOTA-type complexes. Angew. Chem. Int. Ed..

[78] Pollet R., Nair N.N., Marx D. (2011). Water exchange of a ProHance MRI contrast agent: Isomer-dependent free-energy landscapes and mechanisms. Inorg. Chem..

[79] Castelli D.D., Caligara M.C., Botta M., Terreno E., Aime S. (2013). Combined high resolution NMR and ^1^H and ^17^O relaxometric study sheds light on the solution structure and dynamics of the lanthanide(III) complexes of HPDO3A. Inorg. Chem..

[80] Aime S., Barge A., Botta M., De Sousa A.S., Parker D. (1998). Direct NMR spectroscopic observation of a lanthanide-coordinated water molecule whose exchange rate is dependent on the conformation of the complex. Angew. Chem. Int. Ed..

[81] Miller K.J., Saherwala A.A., Webber B.C., Wu Y., Sherry A.D., Woods M. (2010). The population of SAP and TSAP isomers in cyclen-based lanthanide(III) chelates is substantially affected by solvent. Inorg. Chem..

[82] Zhang S., Wu K., Biewer M.C., Sherry A.D. (2001). ^1^H and ^17^O NMR detection of a lanthanide-bound water molecule at ambient temperatures in pure water as solvent. Inorg. Chem..

[83] Marques M.P.M., Geraldes C.F.G.C., Sherry A.D., Merbach A.E., Powell H., Pubanz D., Aime S., Botta M. (1995). NMR conformational study of the lanthanide(III) complexes of DOTA in aqueous solution. J. Alloy. Compd..

